# Recognizing the Universality of Copper Reconstruction Via Dissolution–Redeposition at the Onset of CO_2_ Reduction

**DOI:** 10.1021/acs.jpclett.5c01974

**Published:** 2025-09-04

**Authors:** Blaž Tomc, Marjan Bele, Matic Plut, Mitja Kostelec, Stefan Popović, Mohammed Azeezulla Nazrulla, Francisco Ruiz-Zepeda, Ana Rebeka Kamšek, Martin Šala, Adam Elbataioui, Lidija D. Rafailović, Yasmin Bastos Pissolitto, Francisco Trivinho-Strixino, Wojciech Jerzy Stępniowski, Luka Suhadolnik, Nejc Hodnik

**Affiliations:** a Department of Materials Chemistry, National Institute of Chemistry, Ljubljana 1000, Slovenia; b University of Nova Gorica, Nova Gorica 5000, Slovenia; c Photonic Ensemble Nallur, Karnataka 577221, India; d Institute of Metals and Technology, Ljubljana 1000, Slovenia; e Department of Analytical Chemistry, 68913 National Institute of Chemistry, Ljubljana 1000, Slovenia; f Department of Material Science, Chair of Material Physics, Montanuniversität, Leoben 8700, Austria; g Erich Schmid Institute of Materials Science, Austrian Academy of Sciences, Leoben 8700, Austria; h Institute of Materials Science & Engineering, 69698 Military University of Technology, Warsaw 00908, Poland; i Department of Physics, Chemistry and Mathematics, 613995 Federal University of São Carlos, Sorocaba 18052-780, Brazil

## Abstract

The electrochemical CO_2_ reduction (ECO_2_R) on copper (Cu) remains one of the most promising pathways to convert CO_2_ into value-added products. However, it suffers from severe restructuring, resulting in the unknown structural identity of the ECO_2_R active catalyst. Here, we show that dissolution–redeposition is the universal early-stage restructuring mechanism in ECO_2_R, occurring across all the tested Cu morphologies, including foils, nanoparticles, oxide-derived films, and gas diffusion electrodes. Using identical location scanning electron microscopy, we directly visualize and confirm that this transformation begins at the reaction onset, reshaping catalyst morphology and complicating structure–activity interpretations. Our findings demonstrate that all the Cu catalysts act as precursors to their true, *in situ*-formed active phase, generated through the reduction of Cu oxides and electrolyte-driven dissolution-redeposition. Recognizing the universality of this transformation is essential for accurate mechanistic understanding and the rational design of future Cu-based ECO_2_R catalysts.

## Graphical abstract

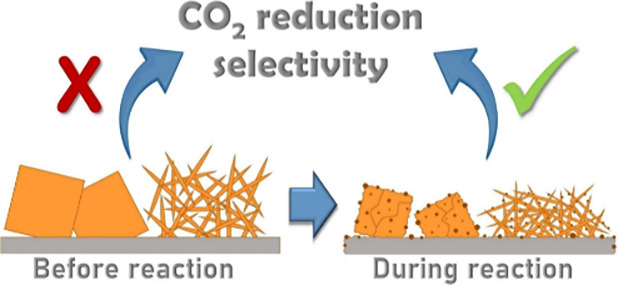

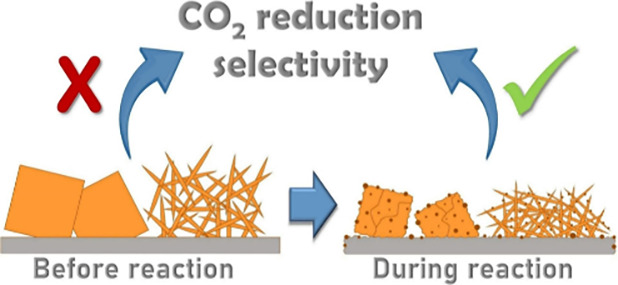

Copper (Cu) based catalysts are central to electrochemical CO_2_ reduction (ECO_2_R) due to their unique ability to convert CO_2_ into multicarbon products such as ethylene and ethanol.^1^ The challenge of improving catalytic performance has driven intense investigation into structure–activity relationships, with most studies correlating product selectivity to Cu surface morphology, crystallographic orientation, and nanoscale features.^2–10^ However, recent studies increasingly question the structural stability of these catalysts under operating conditions.^8–33^

A growing body of evidence shows that Cu surfaces undergo dynamic restructuring via a dissolution-redeposition mechanism almost immediately upon immersion in electrolyte.^13–15^ Cu oxides, virtually unavoidable due to air exposure, partially dissolve under open-circuit conditions.^13–18,34^ Upon the application of cathodic potentials, these dissolved Cu species redeposit, forming new structures that are often unrecognizable from the original ones (Figure 1a).^13–15,19,20^ These early stage changes are shaped by the local environment, including potential and emerging adsorbates like *CO.^15,16,19,20^ As a result, a population of roughened, low-coordinated sites is formed.^4,8,9^ ^,^ ^13,14,19,22^ This self-reconstructed surface represents the actual active Cu surface in the ECO_2_R, dictating the catalytic behavior.^8^

**Figure 1:**
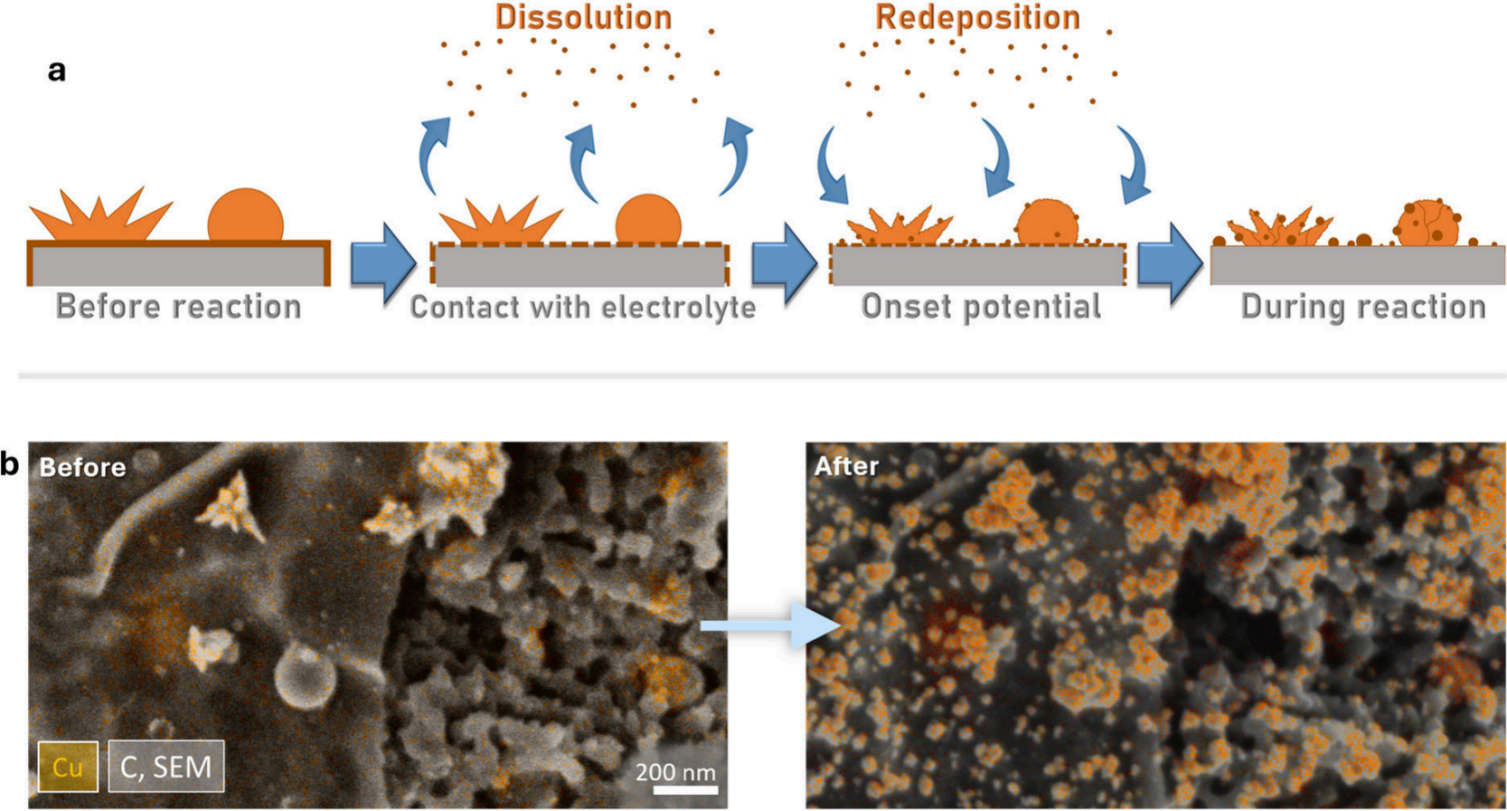
a) Combined proposed mechanism of Cu-based catalysts restructuring at the onset of ECO_2_R, including first the dissolution of Cu into the electrolyte, followed by redeposition at the reaction onset.^13–15^ b) IL-SEM energy-dispersive spectroscopy (EDS) mapping of Cu-based catalysts during the initial stage of ECO_2_R, showcasing a redistribution and restructuring of Cu domains.

Interestingly, ECO_2_R-induced reconstruction has been observed across a wide range of Cu surface morphologies, including nanoparticles, foils, electrodeposited films, oxide-derived Cu (OD-Cu), and gas diffusion electrodes (GDE).^8,13–16,23^ However, the mechanisms underlying these morphological changes have not been fully understood and have therefore not been conclusively interpreted.^8,9^ Moreover, it remains unclear at which stage of the reaction this restructuring occurs, whether it is triggered at the initial onset, at lower potential limits, after repeated cycling, or because of specific material properties or different electrolyte compositions used in various studies. Such variables are often poorly controlled and insufficiently understood. Additionally, assigning ECO_2_R performance to the catalyst’s initial morphology is misleading. Dissolution-redeposition is often completely neglected in ECO_2_R studies in favor of presenting good activity and/or selectivity. The inherent challenge arises since the genuine structure–activity relationships may be wrongly interpreted, since the performance is assigned to the precursor. This leads to misattributions of the observed catalytic performance, which could be a big setback for future catalyst design. To advance the field, it is essential to recognize and incorporate this dynamic restructuring when interpreting structure–activity relationships.

To explore the universality of Cu restructuring at the onset of ECO_2_R, we employed identical location scanning electron microscopy (IL-SEM) to visualize morphological changes across diverse Cu-based catalysts: (i) Cu nanostructures embedded in a carbon matrix (Figure 1b), (ii) two different Cu nanoparticle ensembles on a glassy carbon substrate (Figure 2a,b), (iii) smooth polycrystalline Cu foil (Figure 2c), (iv) OD-Cu films (Figure 2d), (v) Cu on a GDE (Figure 2e), and identical location scanning transmission electron microscopy (IL-STEM) on (vi) Cu nanowires with carbon shells (Figure 2f). The extent and nature of restructuring, ranging from surface roughening to nucleation of new nanostructures, varied depending on morphology, composition, and electrochemical conditions. Despite these differences, a unifying feature across all systems was a change in surface architecture within the onset of the ECO_2_R process.

**Figure 2:**
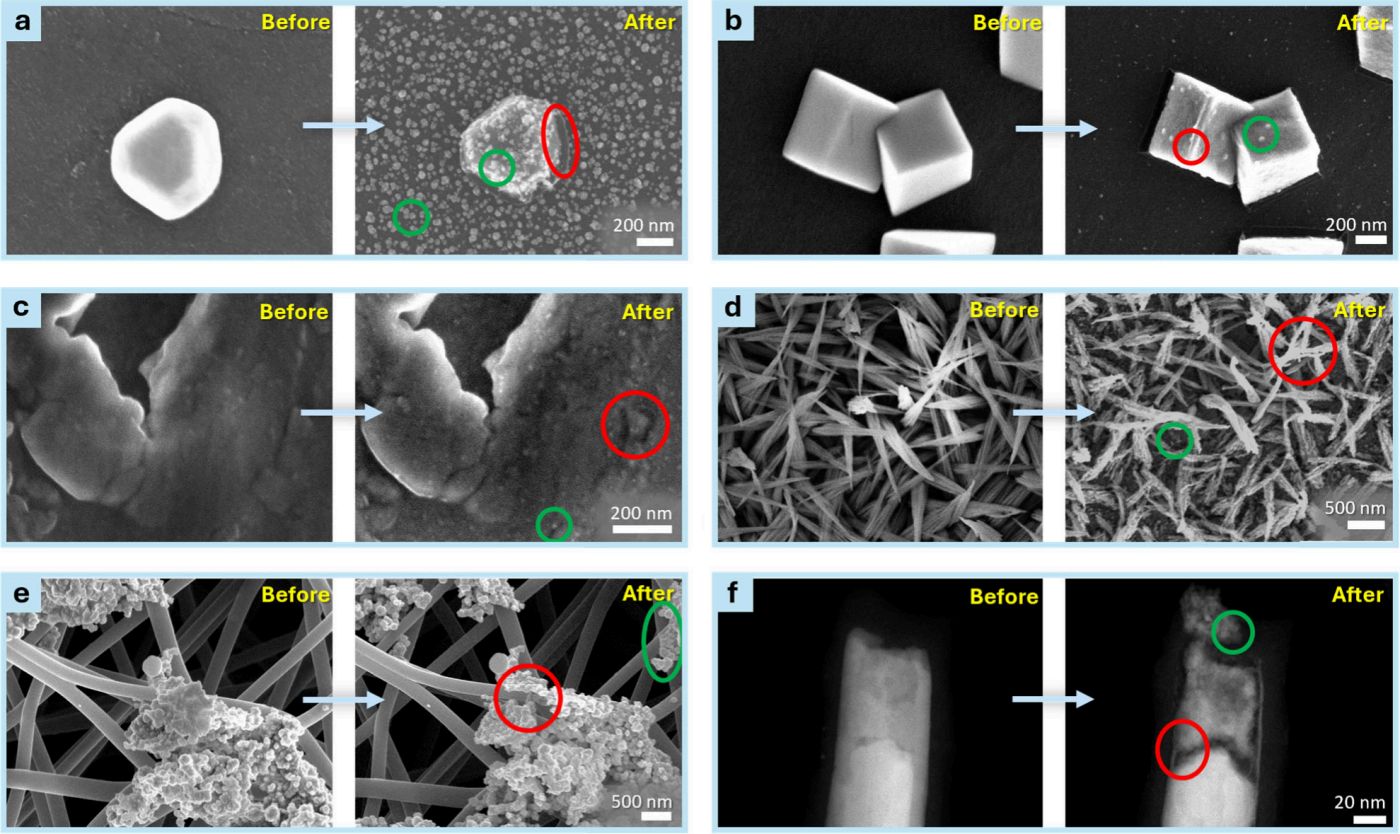
a,b) IL-SEM image of Cu nanoparticles on a glassy carbon substrate showing surface roughening and secondary particle nucleation after electrochemical treatment. c) IL-SEM of polycrystalline Cu foil reveals nanostructuring and increased roughness. d) IL-SEM of OD-Cu structures indicates substantial restructuring via dissolution–redeposition, resulting in pronounced surface changes. e) Aggregated Cu nanostructures deposited on an electrospun gas diffusion layer (GDL) demonstrate Cu morphological evolution in a different setup. f) IL-STEM image of a carbon-coated Cu nanowire shows partial dissolution and formation of new features via redeposition. Across all systems, red highlights mark regions of Cu dissolution while green denotes features formed via redeposition. Lower magnification images of c,d) are presented in Figure S3.

Across all catalyst configurations (Figure 1b and Figure 2), newly formed Cu structures were observed to nucleate across the catalyst surface rather than through the direct growth of pre-existing features (as seen clearly in Figure 2a). This suggests that the initial Cu surface becomes rapidly covered or passivated by the ECO_2_R, likely by early adsorbed intermediates such as CO*,^35–39^ which suppresses further growth at those sites. Therefore, redeposition favors less hindered regions, leading to the emergence of new morphological features across the substrate. Image analysis of Figure 2a (Figure S1) revealed that while major transformations occur, no Cu is lost during the process.

The degree of restructuring is strongly correlated with the initial oxidation state of the catalyst. Cu foil (Figure 2c), containing only a native oxide layer formed in air (EDS determined molar ratio: ∼ Cu_16_O), showed relatively limited surface roughening. In contrast, OD-Cu (Figure 2d), with an engineered oxide film (EDS-determined molar ratio: ∼ CuO_1.7_), underwent substantial collapse and reconstruction. These results highlight the critical role of initial Cu oxide content in determining the magnitude of restructuring. By measuring the open circuit potential (OCP) of the 30 min dissolution of these two catalysts (Figure S2), it is evident that the dissolution under these conditions is thermodynamic.^17^ However, the OCP values of OD-Cu coincide with the potential of the highest measured dissolution, while for the Cu foil, the OCP values are in the region of low dissolution.^17^ To further support this, the electrolytes were analyzed using inductively coupled plasma mass spectrometry (ICP-MS), which determined a 2.7-fold higher concentration of Cu in the electrolyte exposed to OD-Cu (724.3 μg/L) compared to the Cu foil (271.6 μg/L).

To confirm that the early restructuring mechanism indeed affects catalysts’ performance, we systematically compared samples subjected to two different OCP pretreatments and three different onset potentials (Figure 3). These two parameters were chosen as variables since previous studies indicated distinct Cu restructuring by changing them.^15,40^ Substantial restructuring was evident in all examples, but the extent and nature of this transformation varied significantly depending on the pretreatment and applied current (Figure 3). Catalysts exposed to a 600 s OCP period before polarization showed more pronounced morphological changes, with newly formed nanostructures being larger and more uniformly distributed. This contrasts with the samples polarized immediately (i.e., without OCP), where finer and more densely packed structures were observed. The smaller features in the non-OCP condition suggest that dissolution and redeposition occurred only transiently.^15,17^ This distinction indicates two possible effects: (i) more Cu is dissolved during the OCP phase, providing a larger reservoir for redeposition once polarization begins, and (ii) transient dissolution–redeposition occurring under polarization proceeds more rapidly and locally, likely because the dissolved Cu species remain close to the surface before redepositing.

**Figure 3:**
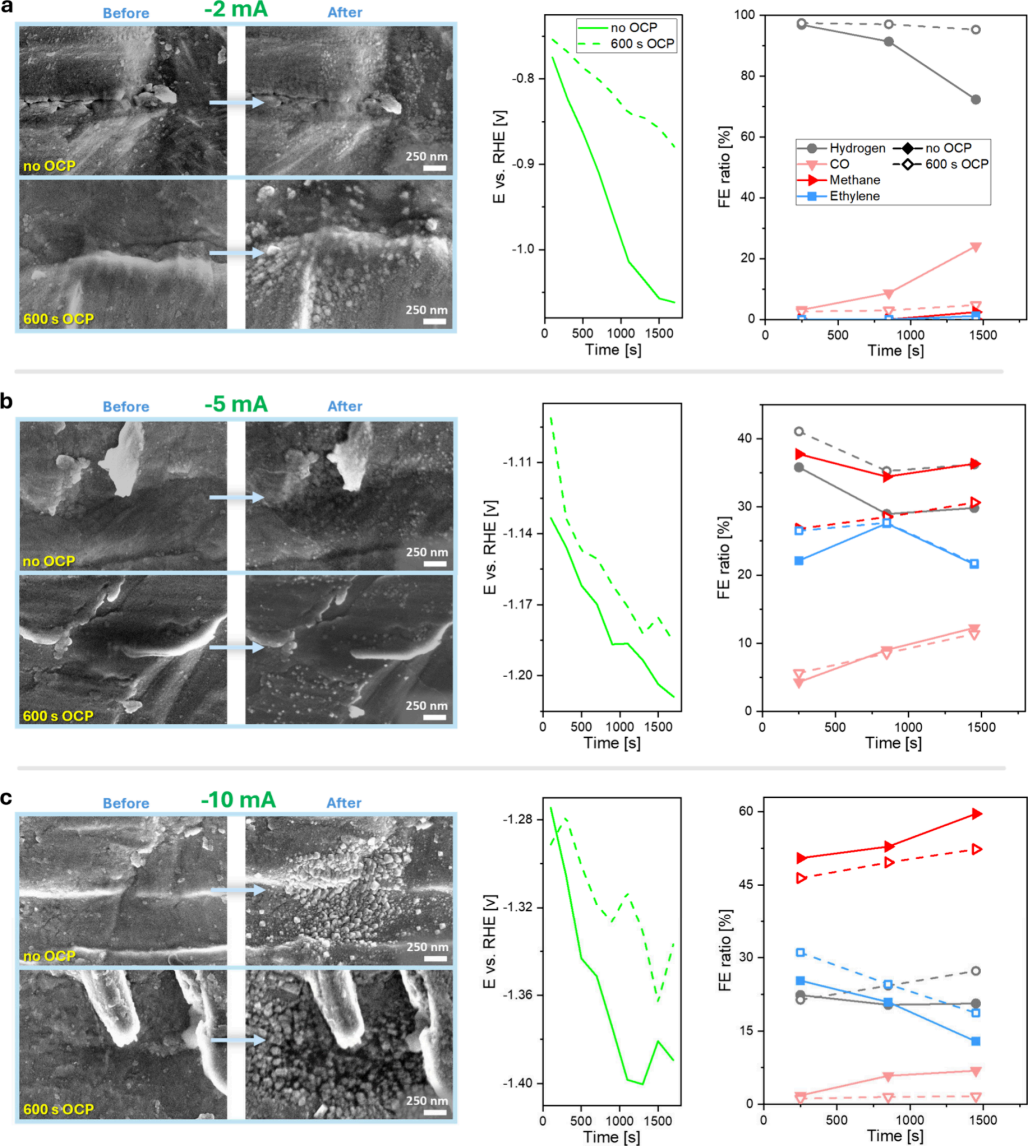
a-c) Morphological and electrochemical evolution of Cu catalysts under different pretreatment conditions and applied current densities. IL-SEM imaging depicts the distinct restructuring of Cu foil at different pretreatments and applied electrochemical biases. These morphological changes correspond to measurable differences in electrochemical behavior: (i) OCP-treated catalysts require lower overpotentials for achieving the same current, and (ii) GC data reveal that OCP pretreatment enhances ethylene and hydrogen selectivity while suppressing CO and methane formation. Total faradaic efficiencies (FE) can be found in Figure S4.

Importantly, electrochemical measurements revealed that OCP-treated samples exhibited higher intrinsic activity (Figure 3). For a given applied current, these samples required a lower overpotential, reflecting improved charge transfer efficiency or better-formed active sites. This correlates well with the observed morphological alterations, which suggest greater surface renewal and restructuring when OCP is included. Gas chromatograph (GC) analysis further clarified the impact of pretreatment, revealing that OCP-treated samples exhibited a distinct selectivity profile compared to non-OCP-treated samples (Figure 3). Catalysts polarized immediately, without OCP, produced less hydrogen but also less ethylene, while improving the selectivity toward CO and methane.

While the findings in Figure 3 clearly indicate that the active sites have changed, they do not directly explain why the reactivity of the Cu surface has shifted. To investigate this, ECO_2_R was performed under two different pretreatment conditions at a constant potential of – 1.025 V vs reversible hydrogen electrode (RHE), with operando electrochemical impedance spectroscopy (EIS) measurements (Figure S5).^41^ These experiments revealed that (i) the current density is higher when OCP pretreatment is applied, (ii) ECO_2_R selectivity differs between the two conditions, (iii) the charge transfer resistance (R_CT_) is significantly lower for the OCP-pretreated sample, and (iv) the electrochemical surface area (ECSA) increases only slightly. Since the ECSA remained comparable while R_CT_ decreased substantially, this indicated that the specific activity of individual sites improved with OCP pretreatment.

The results in this letter confirm that the dissolution–redeposition mechanism is a fundamental restructuring pathway for Cu catalysts during the onset of ECO_2_R, occurring across a range of Cu configurations (Figure 1b and Figure 2). Importantly, the transformation begins at ECO_2_R onset, underscoring that the ″active″ structure is not defined solely by the as-prepared catalyst, but also by the onset environment of the reaction (Figure 3).

The extent of restructuring is closely tied to surface composition and pretreatment. Cu oxides, particularly those formed by anodization or air exposure, dissolve readily when in electrolyte contact. Their subsequent redeposition under electrochemical bias generates entirely new surface features. These changes are more pronounced when a longer OCP is applied (Figure 3), indicating that the diffusion length of dissolved species away from the electrode and their interaction with the evolving applied potential and intermediate coverage affect the final structure. Moreover, the observation that new Cu nanostructures consistently form away from the original features aligns with prior findings that early adsorbed intermediates, such as *CO, can block active sites and steer redeposition elsewhere.^15^ This dynamic explains why the morphology of Cu catalysts often changes drastically within the first few seconds of ECO_2_R.

The electrochemical and ECO_2_R selectivity trends emphasize the nuanced consequences of this restructuring. While the OCP-pretreated Cu foil exhibited improved intrinsic activity and enhanced C_2_ product formation, the simultaneous suppression of C_1_ production and increased hydrogen evolution underscores the dual nature of this restructuring effect, beneficial for certain pathways, but detrimental to others. Such outcomes illustrate that early-stage restructuring is not merely a surface modification but a mechanism that decouples catalytic performance from the as-prepared morphology. Neglecting this transformation risks linking catalytic performance to initial morphologies that are no longer present under operating conditions.

In this work, we demonstrate how a straightforward technique like IL-SEM can be effectively used to observe this dynamic restructuring. By enabling direct before-and-after visualization of catalyst morphology, IL-SEM offers a powerful yet accessible approach to decouple the effects of initial restructuring from intrinsic catalytic behavior.^14,15,42–45^ Its broader adoption could help unify experimental protocols and interpretation strategies across the community, ultimately accelerating mechanistic insight and rational catalyst design in ECO_2_R.

Understanding and incorporating this restructuring mechanism is critically important for interpreting OD-Cu performance, which has shown a high interest in the literature due to its good ECO_2_R catalytic activity.^8–10^ The high oxide content causes major restructuring, as shown in the literature^8,9^ and in this study (Figure 2d). Therefore, attributing the catalytic properties to the initial Cu catalysts in the OD-Cu ECO_2_R studies would evidently be wrong. Only through operando analysis^8,9^ or precise before-and-after comparisons (Figure 2d), some correlations between structure and high selectivity would be comprehended.

While the dissolution–redeposition mechanism poses a challenge for establishing reliable structure–activity relationships, it also presents a unique opportunity: the inherent activation^46^ and even the regenerability of Cu catalysts. Pulsed electrolysis strategies, particularly those incorporating oxidative potentials, can be intentionally designed to capitalize on this dynamic behavior.^31,35,47–52^ By tuning the pulse duration and initial polarization conditions, it is possible to control the Cu morphology restructuring^47^ toward forming fresh, active surface features. This dynamic regeneration could help counteract performance losses by dynamic Cu reconstruction during long-term protocols.^15^ In this light, dissolution–redeposition is not solely a degradation pathway, but a controllable restructuring tool that, if harnessed correctly, could extend catalyst lifetime and sustain high selectivity during extended ECO_2_R. The results from Figure 3 showcase how the applied current (potential) and the time of OCP affect the Cu restructuring that, in turn, affects ECO_2_R selectivity and activity, elucidating previous pulsed electrolysis reports.^47,53^

In conclusion, Cu dissolution–redeposition is recognized as the universal restructuring mechanism in electrochemical CO_2_ reduction, instantly reshaping every Cu catalyst and resetting its activity from the first moment of electrolysis. Identical-location SEM captured this real-time transformation across a set of Cu-based catalysts with various morphologies and revealed that its extent scales with the catalyst’s initial oxidation state: a minimally oxidized Cu foil experienced only a modest surface roughening, whereas oxide-derived Cu with an engineered oxide film collapsed and rebuilt dramatically. These findings demonstrate that steady-state surfaces are a myth during ECO_2_R operation, emphasizing that reliable structure–activity models and rational catalyst design must account for dynamically evolving catalyst surfaces. Harnessing and ultimately steering this transient behavior—e.g., via pulsed electrolysis—will unlock the next generation of operando catalyst engineering.

## Experimental Section

Cu nanocubes and nanoparticles were prepared as described in refs,^14,40^ OD-Cu was prepared via 10 min of electrochemical oxidation of Cu foil in 1.0 M NaOH (POCH, 98.8%) at 100 mV vs Hg|HgO,^54^ carbon-embedded Cu catalyst was prepared as described in ref,^15^ Cu foil was used as received, GDE was prepared by dropcasting a Cu (Cu Nano Powder, Suzhou Canfuo Nanotechnology) ink^15^ onto an electrospun mesh,^55^ and carbon-coated Cu nanowires were synthesized by a solution-based method involving thermal decomposition of Cu salts in the presence of oleylamine and oleic acid, followed by controlled growth under an inert atmosphere.^56^

Catalysts were mounted in a gastight, self-made, “sandwich-type” 3-electrode electrochemical cell made of Teflon.^15^ The reference electrode was Leak-Free Ag/AgCl (Alvatek), and platinum foil was used for the anode. The Selemion membrane separated the cathodic and anodic compartments, which were both filled with 1.2 mL of 0.1 M potassium bicarbonate (KHCO_3_, 99.7% Honeywell, USA). CO_2_ (99,998% Messer, Austria) was bubbled in a cathodic compartment with a constant flow of 2.8 g/h. For the GDE experiments, a conventional cell was used, with a RHE (HydroFlex, Gaskatel) serving as a reference and platinum foil as the counter electrode. The electrolyte was 0.5 M KHCO_3_.

Electrochemical measurements were conducted by utilizing a PalmSens4 potentiostat. The applied protocols were: 25 min OCP followed by −0.975 V vs RHE for 60 min with 100% IR compensation (Figure 1b), 20 min OCP followed by −0.7 V vs RHE for 50 min with 85% IR compensation (Figure 2a), 25 min OCP followed by −1.2 V vs RHE for 30 min with no IR compensation (Figure 2b), no OCP followed by −1.025 V vs RHE for 10 min with 100% IR compensation (Figure 2c), no OCP followed by −25 mA for 60 min (Figure 2d), 10 min OCP followed by −3 V for 30 min with no IR compensation (Figure 2e), 25 min OCP followed by −1.3 V for 60 min with no IR compensation (Figure 2f).

Gas products were analyzed online every 10 min using an SRI 8610C GC equipped with flame ionization and thermal conductivity detectors. Liquid products were analyzed after the reaction with a Bruker AVANCE NEO 400 MHz NMR spectrometer equipped with a 5 mm BB­(F)O Iprobe or BBI probe at 25 °C. The calculated FE’s for gas products were normalized to 100% in the article, with total FE provided in the Supporting Information.

IL-SEM imaging was performed using a ThermoFisher Apreo 2S microscope (Thermo Fisher Scientific, The Netherlands) equipped with an EDS detector, Ultim Max 100 (Oxford, UK). Images were acquired using the T2 in-lens detector under conditions optimized for backscattered electron contrast. IL-STEM was performed on a JEOL ARM 200 CF microscope operated at 80 kV.

Cu was determined in the electrolyte after 30 min of OCP with ICP-MS after 10-fold dilution. For sample dilution and preparation of standards, ultrapure water (Milli-Q, Millipore) and HNO_3_ (Merck, Suprapur) were used. Standards were prepared in-house by dilution of certified, traceable, ICP grade single-element standards (Merck Certipur). An Agilent quadrupole ICP-MS instrument (Agilent 7900, Agilent Technologies, Santa Clara, CA), equipped with a MicroMist glass concentric nebulizer and a Peltier-cooled, Scott-type spray chamber, was used for the measurement.

## Supplementary Material



## Notes

Additional SEM images, image analysis, OCP measurements, liquid products distribution, and operando EIS experiment (PDF)
