# Supplementary material for: Recognizing the Universality of Copper Reconstruction Via Dissolution–Redeposition at the Onset of CO2 Reduction
Source: J Phys Chem Lett. 2025 Sep 4;16(36):9553–60. doi: 10.1021/acs.jpclett.5c01974 (PMC12434722; doi:10.1021/acs.jpclett.5c01974)
Supplement: Supplementary file 1 [file jz5c01974_si_001.pdf]

Supporting Information

Recognizing the Universality of Copper Reconstruction via Dissolution–Redeposition at the Onset of CO_2 Reduction

Blaž Tomc[ab]*, Marjan Bele[a]*, Matic Plut[a], Mitja Kostelec[ab], Stefan Popović[a], Mohammed Azeezulla Nazrulla[ac], Francisco Ruiz-Zepeda[ad], Ana Rebeka Kamšek[a], Martin Šala[e], Adam Elbataioui[f], Lidija D. Rafailović[g], Yasmin Bastos Pissolitto[hi], Francisco Trivinho-Strixino[i], Wojciech Jerzy Stępniowski[h], Luka Suhadolnik[a], Nejc Hodnik[abd]*

[a]Department of Materials Chemistry, National Institute of Chemistry, Ljubljana 1000, Slovenia

[b]University of Nova Gorica, Nova Gorica 5000, Slovenia

[c]Photonic Ensemble Nallur, Karnataka 577221, India

[d]Institute of Metals and Technology, Ljubljana 1000, Slovenia

[e]Department of Analytical Chemistry, National Institute of Chemistry, Ljubljana 1000, Slovenia

[f]Department of Material Science, Chair of Material Physics, Montanuniversität, Leoben 8700, Austria

[g]Erich Schmid Institute of Materials Science, Austrian Academy of Sciences, Leoben 8700, Austria

[h]Institute of Materials Science & Engineering, Military University of Technology, Warsaw 00908, Poland

[i]Department of Physics, Chemistry and Mathematics, Federal University of São Carlos, Sorocaba 18052-780, Brazil

email: *Nejc Hodnik: nejc.hodnik@ki.si, *Marjan Bele: Marjan.bele@ki.si, *Blaž Tomc: blaz.tomc@ki.si

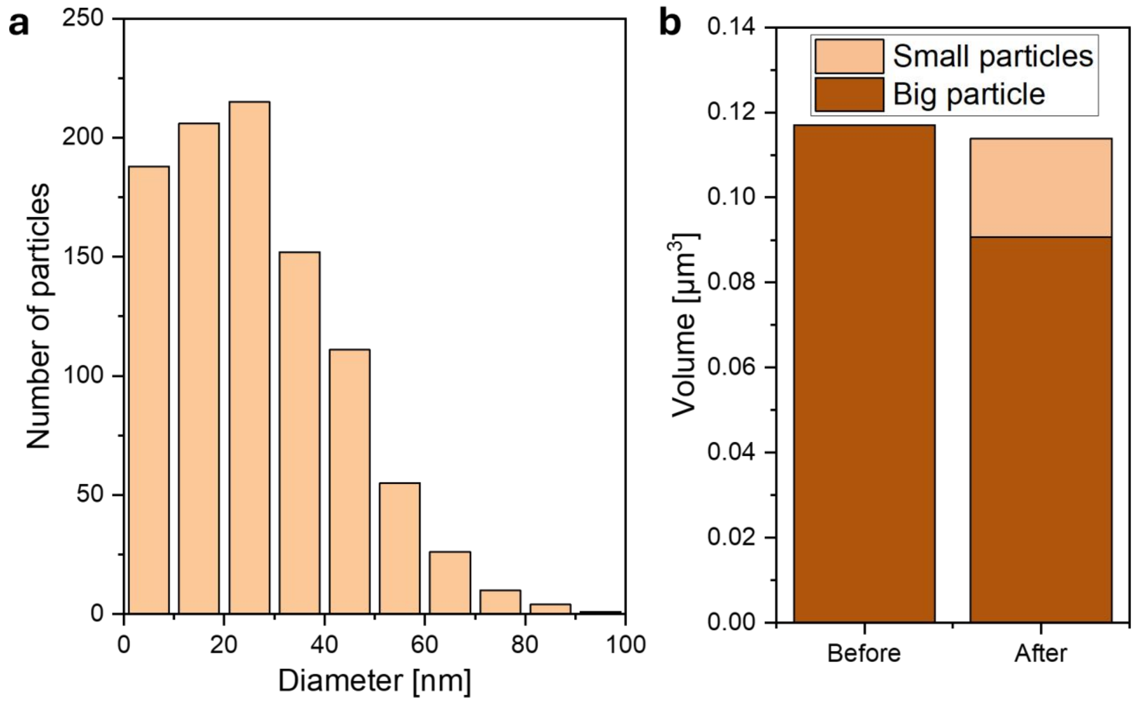

Figure S1: Image analysis of Figure 2a with a thresholding feature in ImageJ. a) Nanoparticle diameter distribution of the image after reaction. b) The approximated volume of the nanoparticles before and after electrochemical CO_2 reduction (ECO_2R). The before image consists of only one particle with a diameter of 607 nm, while the after image consists of a partially dissolved nanoparticle with a diameter of 558 nm and smaller nanoparticles with diameters depicted in (a). The volumes were calculated as a perfect sphere for rough estimation.

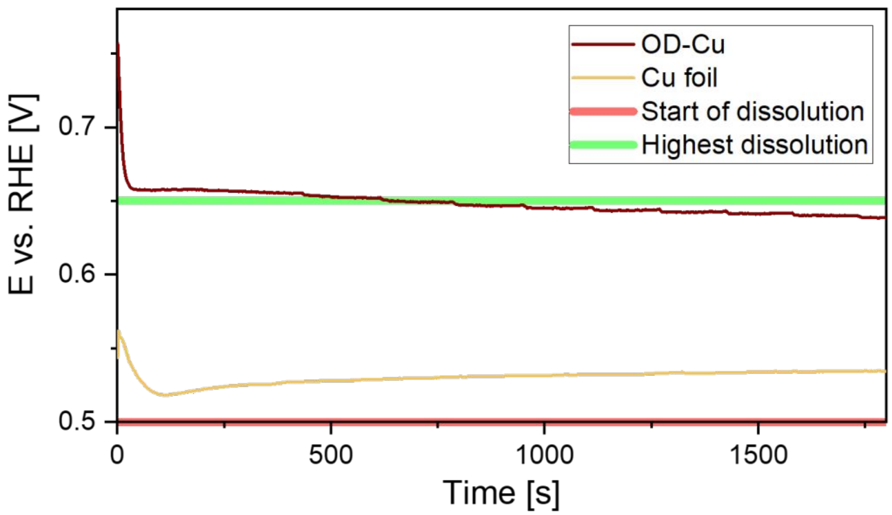

Figure S2: Open circuit potential (OCP) value of 30-minute electrolyte exposure of two differently oxidized Cu catalysts. The reference lines are from [17], annotating at which potential Cu dissolution starts at pH 6.8, and where it is the most pronounced.

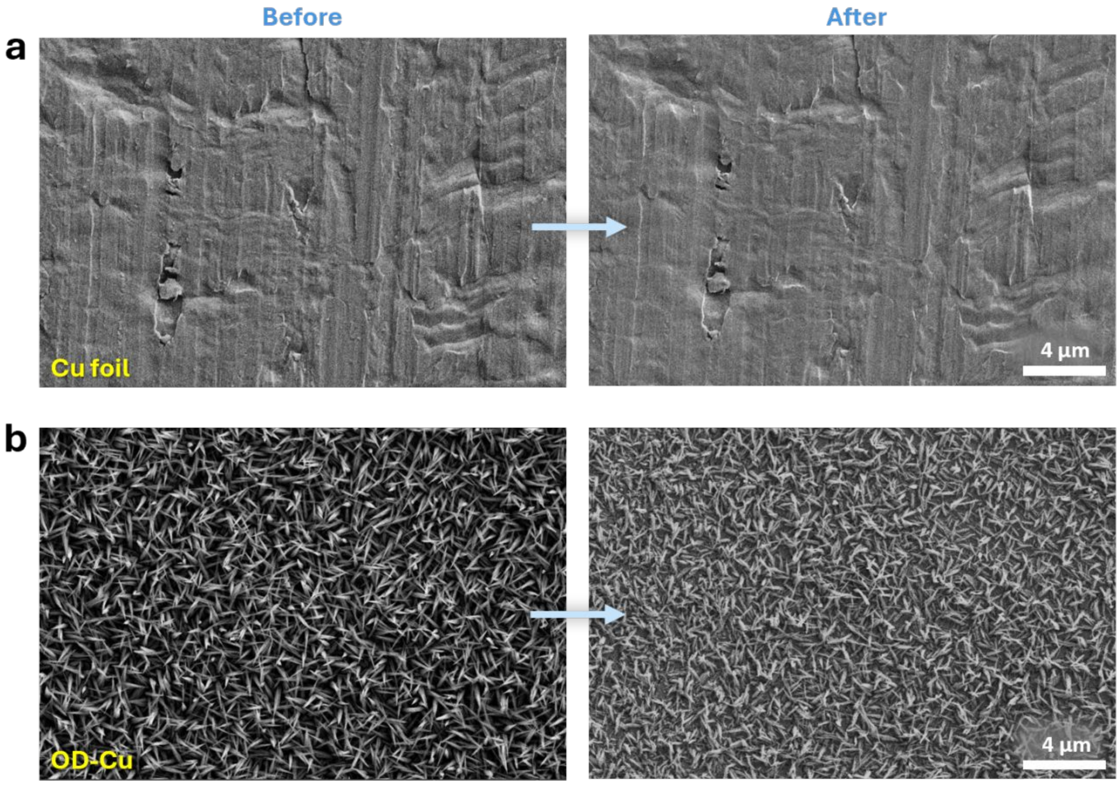

Figure S3: Lower magnification images of Figure 2c,d.

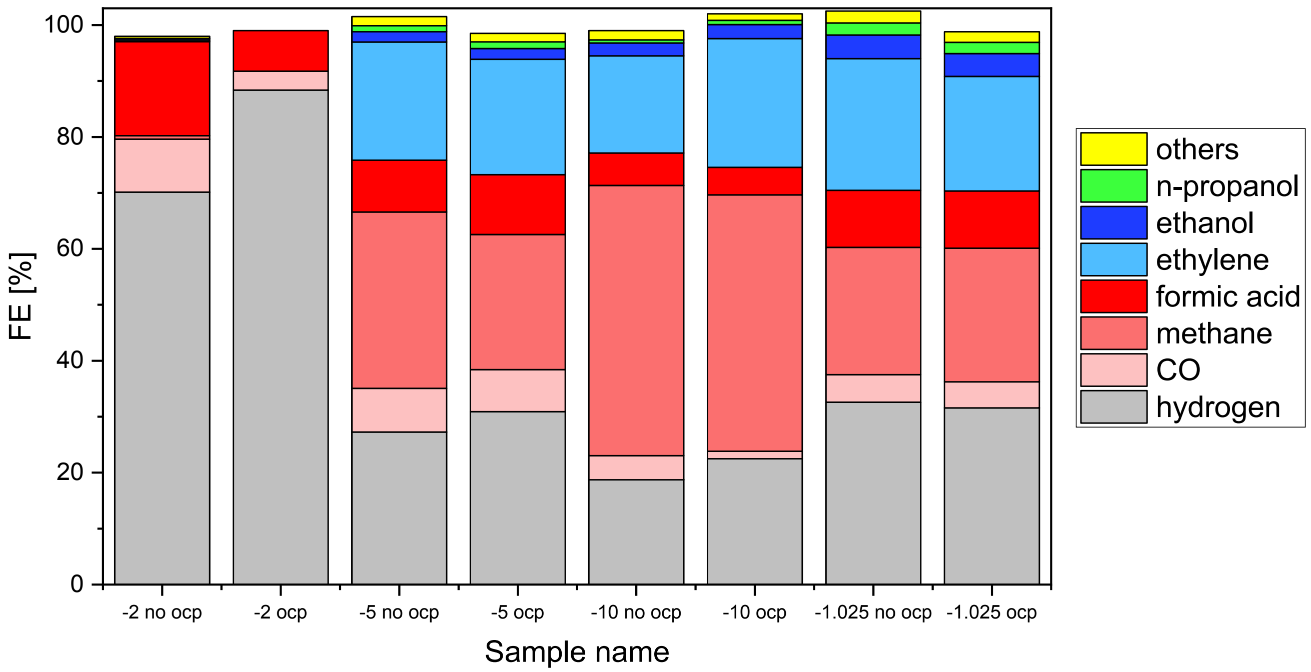

Figure S4: Total faradaic efficiency (FE) for ECO_2R products distribution for the experiments in Figure 3 and Figure S5a.

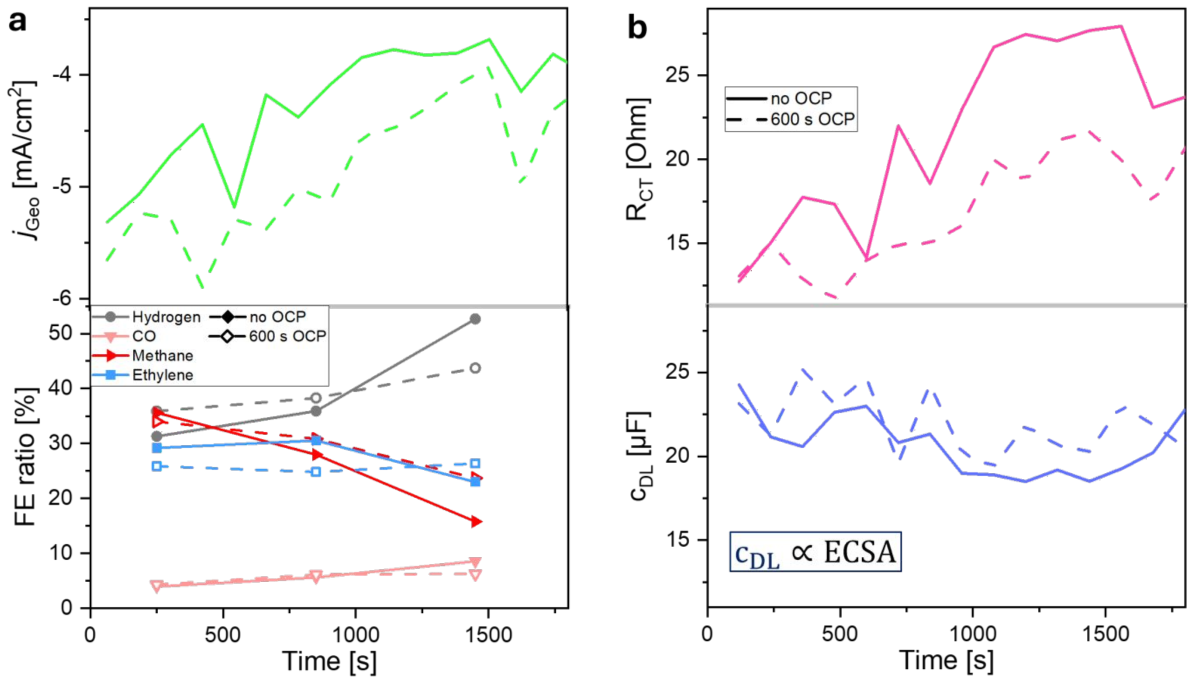

Figure S5: ECO_2R at Cu foil measured with operando electrochemical impedance spectroscopy (EIS)[41] at -1.025 V vs. RHE at two different pretreatment conditions. With this experiment, the underlying mechanisms for the different ECO_2R efficiencies were measured. The key to meaningful data lies in EIS being measured at exact reaction conditions, as presented in [41]. Therefore, a) activity and selectivity could be directly connected to b) the charge transfer resistance (R_{CT}) and double layer capacitance (c_{DL}). The c_{DL} parameter is scaled with electrochemical surface area (ECSA).
